# The Psychology of the Emotions

**Published:** 1898-09

**Authors:** 


**The Psychology of the Emotions.** By TH. RIBOT. Pp. xix., 455. London: Walter Scott, Ltd. 1897.

Ribot expounds through this book his views as to the essential nature of states of the feelings. He adopts the physiological thesis unreservedly; that is, that these states are primary and independent of intelligence, are connected with biological conditions and are the direct expression of vegetative life, having their root in movements.

The plan of the work is well conceived; general manifestations of feeling and especial emotions are studied separately and, with obvious advantage to a clear understanding of the subject, each in order of evolution and dissolution.

It would be impracticable within any moderate limit to discuss this book in detail; all that is needful is to pay tribute to it as a production praiseworthy from every point of view. The author's power of lucid, concise, illustrative language is recognised and is herein shewn at its best; there is neither a dull nor a superfluous page, whilst at the same time every opinion stated gives evidence of its careful and dispassionate consideration.

Here is, therefore, a volume of the greatest value to all interested in the study of mind, and those portions devoted to pathology must prove of especial assistance to workers in mental disease. The careful printing is suitable complement to the admirable subject-matter.

